# Expression and Function of Neuron-Glia-Related Cell Adhesion Molecule (NrCAM) in the Amygdalar Pathway

**DOI:** 10.3389/fcell.2019.00009

**Published:** 2019-01-31

**Authors:** Vishwa Mohan, Julia R. Gomez, Patricia F. Maness

**Affiliations:** Department of Biochemistry and Biophysics, University of North Carolina School of Medicine, Chapel Hill, NC, United States

**Keywords:** stria terminalis, NrCAM, bed nucleus of the stria terminalis, fear conditioning, limbic system

## Abstract

Neuron-Glia related cell adhesion molecule (NrCAM) is a candidate autism risk factor that promotes axon guidance through cytoskeletal linkages in developing brain but its role in limbic circuitry has not been investigated. *In situ* hybridization (ISH) and immunofluorescence staining showed that NrCAM is expressed in the developing amygdalar pathway of mouse embryos during outgrowth of projections in the stria terminalis, a major limbic tract that interconnects the central amygdala (CeA) with key targets in the bed nucleus of the stria terminalis (BNST). Analysis of fiber tracts in NrCAM mutant mice by Neurofilament protein immunohistochemistry showed pronounced defasciculation and misprojection of fibers in the ST. The defasciculation phenotype may result from impairment in NrCAM homophilic inter-axonal adhesion or axon repulsion from the secreted ligand Semaphorin 3F, which is expressed in limbic areas in proximity to the ST. Behavioral testing indicated that NrCAM null mice were impaired in context-dependent fear conditioning, in accord with altered amygdala-BNST connectivity, but displayed normal cued (tone-shock) conditioning. Results are consistent with the novel finding that NrCAM mediates fasciculation of axon fibers in the ST important for proper amygdalar-BNST circuitry and response to contextual fear conditioning.

## Introduction

During brain development a diversity of guidance cues and receptors is required for correct synaptic targeting of axons. One important group of guidance receptors is represented by immunoglobulin-class (Ig) cell adhesion molecules of the L1 family (L1-CAMs) (Sytnyk et al., 2017). Among these, Neuron-Glia related cell adhesion molecule (NrCAM), L1, and Close Homolog of L1 promote axon growth through *trans* homophilic binding of their extracellular domains and intracellular coupling of their conserved cytoplasmic domains to the actin cytoskeleton. Cytoskeletal coupling of L1-CAMs is achieved through direct binding to the actin adaptors Ankyrin and Ezrin-Radixin-Moesin proteins (ERM), as well as to PDZ-containing scaffold proteins PSD-95 and SAP102 (Buhusi et al., 2008; Schlatter et al., 2008; Demyanenko et al., 2014; Leshchyns’ka and Sytnyk, 2016). NrCAM is notable as a potential target for mutation in neurodevelopmental disease, as polymorphisms in the NrCAM locus have been associated with autism spectrum disorders (ASD) (Pinto et al., 2010; Voineagu et al., 2011; Sakurai, 2012). Moreover, male NrCAM knockout mice exhibit autism-related behaviors, including impaired sociability, cognitive rigidity, and repetitive behavior (Moy et al., 2009a).

**FIGURE 1:**
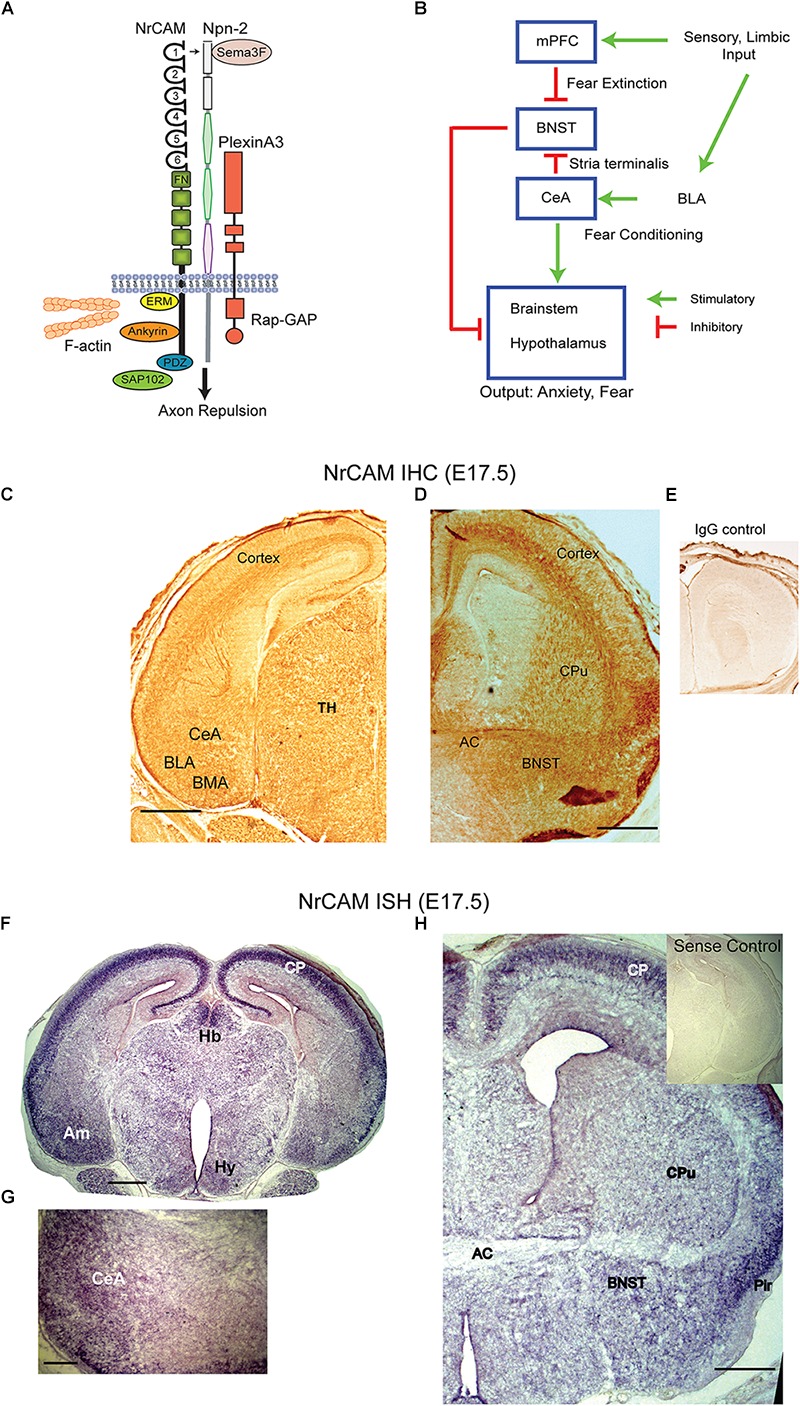
NrCAM expression in the amygdalar pathway. **(A)** Molecular model of Sema3F holoreceptor complex. NrCAM interacts with the Sema3F co-receptor Npn-2, which binds PlexinA3 with Rap-GTPase activating protein (Rap-GAP) activity, leading to axon repulsion. The NrCAM cytoplasmic domain recruits actin cytoskeletal adapters Ezrin-Radixin-Moesin (ERM), Ankyrin, and PDZ-interacting scaffold protein SAP102. **(B)** Schematic of cortico-limbic connectivity. Sensory and limbic input is received by the basolateral amygdala (BLA). The BLA promotes fear conditioning by connections with the central amygdala (CeA). The CeA sends inhibitory GABAergic projections to the BNST and stimulates the brainstem and hypothalamus. Input to the mPFC promotes fear extinction by inhibiting the BNST. **(C)** Immunohistochemical staining (IHC) for NrCAM in coronal brain sections from E17.5 WT mouse brain. NrCAM immunoperoxidase staining is observed in developing nuclei (CeA, BLA, basomedial) of the amygdala, cortex, and thalamus. Scale bar = 1000 μm. **(D)** NrCAM immunoreactivity in E17.5 mouse brain is present in the BNST, cortex, anterior commissure (AC), and caudate putamen (CPu). Scale bar = 1000 μm. **(E)** Control labeling with nonimmune IgG. **(F)** *In situ* hybridization (ISH) of NrCAM mRNA in coronal sections of E17.5 mouse brain shows enrichment in the amygdala (Am), cortical plate (CP), habenula (Hb), and hypothalamus (Hy), Scale bar = 1000 μm. **(G)** Higher magnification showing ISH of NrCAM mRNA in the region of the CeA nucleus of the amygdala at E17.5. Scale bar = 250 μm. **(H)** ISH of NrCAM mRNA in E17.5 mouse brain also shows expression in the BNST adjacent to the anterior commissure (AC), as well as the cortical plate (CP) and piriform cortex (Pir). Scale bar = 1000 μm.

Neuron-Glia related cell adhesion molecule mediates axon repulsion in response to the repellent ligand Semaphorin 3F (Sema3F) (Falk et al., 2005; Demyanenko et al., 2011). In this role NrCAM functions as an integral component of the Sema3F holoreceptor complex, which comprises the co-receptor Neuropilin-2 (Npn2) and signaling subunit PlexinA3 (PlexA3) (Figure 1A). In the presence of Sema3F, NrCAM induces clustering of Npn2 and PlexA3 in the neuronal membrane to activate intrinsic PlexA3 Rap-GAP activity (Mohan et al., 2018). Sema3F-induced axon repulsion through NrCAM is a key mechanism for regulating guidance of thalamocortical (Demyanenko et al., 2011) and commissural axon projections (Falk et al., 2005). In the developing limbic system Sema3F and Npn2 are expressed at discrete locations, where they are required for proper development of the stria terminalis (ST), a nerve fiber bundle that interconnects the central amygdala (CeA) and bed nucleus of the ST (BNST) (Sahay et al., 2003). Connectivity between the CeA and BNST is critical for fear and stress responses, as well as for social interactions (Davis et al., 2010; Coria-Avila et al., 2014; Daniel and Rainnie, 2016; Lebow and Chen, 2016). Because a role for NrCAM in this limbic pathway has not been investigated, we hypothesized that NrCAM may be involved in regulating the development of ST projections between the CeA and BNST, and this might affect behavioral responses to contextual fear conditioning, which depends in part on amygdalar-BNST circuitry (Stamatakis et al., 2014).

To probe this hypothesis, we analyzed NrCAM expression in the amygdalar-BNST projection and studied the effects of NrCAM deletion on the structure and function of this limbic connection in NrCAM null mice. We found that NrCAM was expressed in the ST, CeA, and BNST during establishment of amygdalar connectivity, and that deletion of NrCAM in null mutant mice caused pronounced disruption of axonal tracts in the ST due to defasciculation of axon bundles. Behavioral testing showed further that NrCAM null mice were impaired for contextual fear conditioning. These novel results suggested that NrCAM mediates fasciculation of axons in the ST through homophilic adhesion or repellent guidance, important for contextual fear conditioning responses.

## Materials and Methods

### Mice

Wild type (WT) and homozygous null NrCAM mutant mice were bred for over 10 generations to establish them on a C57BL/6 genetic background. Mice were group-housed in ventilated microisolator cages with free access to water and Prolab RMH 3000 chow. The housing room had a 12-h light/dark cycle (lights off at 7:00 p.m.). Embryonic day E0.5 was defined as the plug date and the day of birth as P0. All animal studies were approved by the Institutional Animal Care and Use Committee (IACUC) of The University of North Carolina School of Medicine at Chapel Hill (IACUC Protocol # 15-114). Mice were handled according to the University of North Carolina IACUC policies and in accordance with NIH guidelines for humane care and use of laboratory animals. All the experiments were performed with male mice except experiments with mouse embryos, which were of mixed gender.

### NrCAM and Neurofilament Immunohistochemistry and Immunofluorescence Staining

Wild type and NrCAM null littermates were anesthetized and perfused transcardially with 4% paraformaldehyde (PFA) buffered in phosphate buffered saline (PBS). For embryos, the head was subjected to fixation without perfusion, and cryo-protected by immersing in 30% sucrose in PBS. Coronal cryostat sections (24 μm) were mounted on slides and stored at -80°C. For immunoperoxidase staining, sections were subjected to antigen retrieval by brief exposure to 10 mM citrate (pH 6) at 100°C. After three washes with PBS, endogenous peroxidase activity was quenched by treating 5 min with 3% H_2_O_2_. After washing with PBS, sections were blocked at room temperature (RT) in PBS containing 5% goat serum and 0.3% Triton X-100. Sections were incubated overnight with monoclonal Neurofilament antibody 2H3 (DSHB, Iowa City, IA, United States) (1:200). For NrCAM sections were incubated with rabbit polyclonal antibody (AbCAM #24344). We and others have used this antibody previously and validated the specificity of this antibody by immunostaining and immunoblotting on WT and NrCAM null samples (Amor et al., 2014; Demyanenko et al., 2014; Morelli et al., 2017; Mohan et al., 2018). Sections were washed with PBS and incubated with biotinylated anti-mouse secondary antibody (1:200, Vector Laboratories) for 1 h at RT. Immunoreactivity was visualized using a Vectastain avidin-biotinylated peroxidase kit. Sections were dehydrated in an ascending series of 50, 70, 90, and 100% ethanol and cleared in xylene before mounting in Permount (Fisher Chemicals). Bright-field images were taken on a Zeiss Axioplan microscope. For quantitative estimation of the degree of fasciculation of the ST, images of Neurofilament-stained coronal sections (24 μm; 3 sections per brain, spanning the entire ST) of WT and NrCAM null mutant brains (P30; 3 mice per genotype) were analyzed at the same magnification and rostro-caudal level using Image J. The line measuring tool was used to obtain the width of the ST at multiple locations along its length, and the average width calculated. The average width of ST in all sections was determined for each mouse, and the mean width per genotype (+SEM) was calculated. Statistically significant differences in the mean width of the ST were analyzed by the *t*-test (two tailed, unequal variances; ^∗^*p* < 0.05).

For double immunofluorescence staining, sections were incubated overnight with Neurofilament antibody 2H3 (1:400) and NrCAM antibody (AbCAM #24344; 1:200). After washing in PBS, sections were treated with anti-mouse AlexaFluor 488 and anti-rabbit AlexaFluor 555 secondary antibodies (1:500, LifeTechnologies) for 1 h at RT. After washing in PBS, sections were mounted on slides using ProLong gold antifade mountant (Invitrogen). Images were obtained using a Zeiss LSM700 confocal microscope at identical settings to compare WT and mutant staining.

### *In situ* Hybridization of NrCAM Transcripts

A pBlueScript (pBS) plasmid containing mouse NrCAM cDNA (GenBank accession number AJ54321, nucleotides 460-1470) was used to generate digoxigenin (DIG)-labeled riboprobes for *in situ* hybridization (ISH) to NrCAM transcripts as described (Demyanenko et al., 2011) in the histology core facility of the UNC Neuroscience Research Center. Briefly, fetal mouse brains (E17.5) were fixed in 4% PFA buffered in PBS overnight at 4°C. Brains were cryo-protected by immersing in an ascending series of 10, 20, and 30% sucrose solution in PBS at 4°C. Coronal sections were cut at 20 μm on a cryostat and mounted onto slides. ISH was performed using digoxygenin-labeled anti-sense and control sense riboprobes as reported (Bartsch et al., 1994; Colbert et al., 1995), and images were captured digitally on a Zeiss Axioplan 2 microscope.

### Behavioral Testing

Adult WT and NrCAM homozygous null littermates (14 WT NrCAM^+/+^ and 20 NrCAM ^-/-^ male mice) were evaluated for fear memory in cued- and contextual fear conditioning tests using the Near-Infrared image tracking system (Med Associates Inc., Burlington, VT, United States) in the UNC Mouse Behavioral Phenotyping Core of the Carolina Institute for Developmental Disabilities (Dr. Sheryl S. Moy, Director). This apparatus has a sound attenuating cubicle with a near infrared/visible lighting system, which has been described and used extensively for fear conditioning in mice and other species (Huang et al., 2013; Zhu et al., 2014). The fear conditioning chamber has a digital video camera and infrared sensors on each side that permit freezing to be recorded by measuring the latency to break infrared beams. Levels of freezing (no movement for 0.5 s) were automatically measured by the image tracking software. The procedure was conducted across 3 days. On the first day, mice were given a 7-min training session. Mice were placed in the test chamber, contained in a sound-attenuating box, and allowed to explore for 2 min. The mice were then exposed to a 30-s tone (80 dB), followed by a 2-s scrambled foot shock (0.4 mA). Mice received two additional shock-tone pairings, with 80 s between each pairing. Context-dependent learning was evaluated on the second day of testing. Mice were placed back into the original test chamber, and levels of freezing (immobility) were determined across a 5-min session. On the third day of testing, mice were evaluated for associative learning to the auditory cue in a final 5-min session. The fear conditioning test chamber was cleaned thoroughly with 70% ethanol before each session. After conditioning, animals were returned to home cages in standard housing. The conditioning chambers were modified using a Plexiglas insert to change the wall and floor surface, and a novel odor (vanilla flavoring) was added to the sound-attenuating box. Mice were placed in the modified chamber and allowed to explore. After 2 min, the acoustic stimulus was presented for a 3-min period. Levels of freezing before and during the stimulus were obtained by the image tracking system. All mice were 6–8 months of age at the time of behavioral testing. A small subset of these mice (3 WT and 5 NrCAM-/-) were tested 6 months previously in a study on oxytocin effects in a social approach task, and were not handled in the interim. No effect of oxytocin on sociability was observed in any of these mice, therefore, it is unlikely that this could affect the observations reported here. Results were analyzed using repeated measures analysis of variance (ANOVAs) with the factor genotype (WT or NrCAM null). Fisher’s protected least-significant difference (PLSD) tests was used for comparing group means only when a significant F value was determined in the overall ANOVA. For all comparisons, significance was set at *p* < 0.05.

## Results

### NrCAM Is Expressed in Limbic Regions of the Embryonic Mouse Brain During Establishment of Amydgalar Connectivity

In cortico-limbic circuitry (Figure 1B) sensory and limbic inputs to the basolateral amygdala promote conditioned fear through connectivity with the CeA ultimately targeting the brain stem and hypothalamus (Hy) to evoke autonomic responses of fear and anxiety. GABAergic neurons in the CeA are notable in making inhibitory connections with the BNST that dampen these responses (Knobloch et al., 2012; Kim et al., 2013). In addition, the medial prefrontal cortex (mPFC) provides “top-down” extinction of responses through connections with the BNST (Amano et al., 2010; John et al., 2013).

To investigate a potential involvement of NrCAM in influencing connectivity between the amygdala and BNST we analyzed NrCAM expression by immunohistochemistry using immunoperoxidase staining, and by ISH in WT mouse embryos at E17.5, when CeA to BNST projections extend and grow toward their target areas (Sahay et al., 2003). Immunoperoxidase staining for NrCAM protein showed widespread NrCAM immunoreactivity in the cortex, thalamus, and amygdalar area (Figure 1C). In the E17.5 amygdala NrCAM was expressed at sites corresponding to central (CeA), basolateral (BLA), and basomedial (BMA) amygdalar nuclei (Figure 1C). NrCAM staining was also seen in the BNST, caudate putamen (CPu), and anterior commissure (AC) (Figure 1D). Control staining with nonimmune IgG was minimal (Figure 1E). NrCAM immunoreactivity marks the location of NrCAM in cell bodies as well as its presence in fibers traversing these regions. To identify the location of NrCAM expressing soma, NrCAM transcripts were localized by ISH in brain sections of WT mice at E17.5. Brain regions were identified by neuroanatomical landmarks and comparison to atlas coordinates for E17.5 mouse brain (Paxinos et al., 2007). NrCAM transcripts were detected by hybridization to an anti-sense riboprobe and found to be abundant in the cortical plate (CP) and amygdala (Am) (Figure 1F). Labeling was also present in the medial habenula (Hb) and Hy. When imaged at higher magnification, NrCAM transcripts were evident in the CeA (Figure 1G). In more rostral sections NrCAM transcripts were present in the BNST adjacent to the AC, as well as the CPu (Figure 1H). High levels of expression are observed in the CP and piriform cortex (Pir). Control hybridization to the sense riboprobe elicited no detectable labeling (Figure 1H, inset). These results showed that NrCAM was expressed in the CeA-BNST pathway during axon targeting in the developing mouse limbic system.

### Axons in the Stria Terminalis Express NrCAM, and Are Defasciculated in NrCAM Null Mice

**FIGURE 2:**
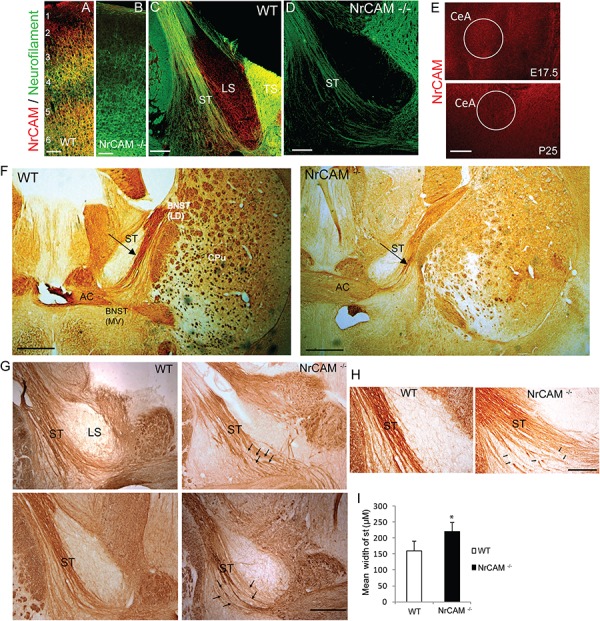
NrCAM expression and effect of deletion on the ST of NrCAM Null Mice. **(A,B)** Representative images of immunofluorescence staining for NrCAM (red) and Neurofilament protein (green) in coronal sections through the brain of P25 WT and NrCAM null mice. Scale bar = 150 μm. **(C)** NrCAM immunofluorescence staining was prominently localized in Neurofilament-positive fibers of the ST in WT mice (P25). NrCAM staining was also present in the lateral and triangular septa (LS, TS). **(D)** NrCAM immunostaining was not detectable in the Neurofilament-positive ST or other regions in this region of the NrCAM null brain (P25). Scale bar = 100 μm. **(E)** NrCAM immunofluorescence staining in the central amygdalar nucleus (CeA) was prominent in WT embryos at E17.5 and declined to lower levels at P25. Scale bar = 100 μm. **(F–H)** Neurofilament immunoperoxidase staining in coronal sections of WT mouse brain at P30. **(F)** Left panel: WT brain showing the ST entering the BNST as a tightly fasciculated bundle. Right panel: NrCAM null brain showing the ST with defasciculated fibers (arrow). Abbreviations: BNST (LD-lateral dorsal and MV-medial ventral areas), anterior commissure (AC), caudate putamen (CPu). Scale bar = 1000 μm. **(G)** Images from different P30 individual mice (upper and lower panels) showing the ST as a well-formed bundle in WT brains, compared to NrCAM null brains, in which ST axons appear defasciculated (arrows). LS, lateral septum. Scale bar = 250 μm. **(H)** High magnification images showing defasciculated and straying ST fibers (arrows) in NrCAM null compared to WT mice at P30. Scale bar = 200 μm. **(I)** Histogram comparing the mean width of the ST in WT and NrCAM null mice (P30), measured in three sections spanning the ST per mouse, as described in Materials and Methods. Mean + SEM; *n* = 3 mice/genotype; ^∗^*p* < 0.05.

To validate the specificity of the NrCAM antibody, double immunofluorescence staining for NrCAM and Neurofilament protein was performed at postnatal day P25 in WT and NrCAM null mutant neocortex, as this region is known to express high levels of NrCAM postnatally (Mohan et al., 2018) (Figure 2A,B). NrCAM immunofluorescence staining was prominent in WT mice as shown in the cortex, whereas it was absent in NrCAM null cortex (Figure 2A,B). Neurofilament immunofluorescence staining of axons was clearly evident in the cortex of both genotypes. To assess the localization of NrCAM in the ST postnatally, we performed immunofluorescence staining for NrCAM and Neurofilament in brain sections through the ST of WT mice at P25. NrCAM immunofluorescence staining was present in Neurofilament-positive fibers of the ST (Figure 2C). NrCAM staining was also evident in the triangular and lateral septum (LS). In contrast, NrCAM immunofluorescence was absent from the ST and adjacent areas in the brain of NrCAM null mice, validating the specificity of the staining (Figure 2D). ST fibers originating in the CeA extend toward the BNST at approximately E17.5 and complete synaptic targeting as development approaches P30 (Sahay et al., 2003). We also assessed the expression of NrCAM in the amygdalar region of WT mice at two key developmental stages, E17.5 and P25, by immunofluorescence staining. Results showed that NrCAM was more prominently expressed in embryos at E17.5 then declined to lower levels postnatally at P25 (Figure 2E).

The ST is a highly fasciculated axon tract in the mature limbic system (Stamatakis et al., 2014). The presence of NrCAM in the ST suggested that homophilic or heterophilic interactions within the extracellular region of NrCAM might play a role in fasciculation of axon bundles comprising the ST. To visualize the ST, we performed immunoperoxidase labeling of Neurofilament protein in mice at postnatal day P30, when the tract is fully formed. In WT mice the ST appeared as a highly fasciculated fiber bundle dorsal to the AC (Figure 2F, left panel). In sections of NrCAM null mutant mice matched for rostrocaudal level, the ST appeared broader in width, with fibers that strayed from the main tract and appeared disorganized (Figure 2F, right panel). At higher magnification of WT brain sections from other mice, ST fibers ran as a relatively tight bundle, whereas in NrCAM null mice fibers appeared defasciculated (Figure 2G,H; arrows). To provide a quantitative estimate of defasciculation, the average width of the ST was measured in serial coronal sections matched for level from WT and NrCAM mutant brains (*n* = 3 mice/genotype), and compared for significant mean differences. This analysis indicated that the mean width of the ST in NrCAM mutant mice was significantly increased compared to WT (*t*-test, ^∗^*p* < 0.05) (Figure 2I).

### Contextual Fear Conditioning Is Impaired in NrCAM Null Mice

**FIGURE 3:**
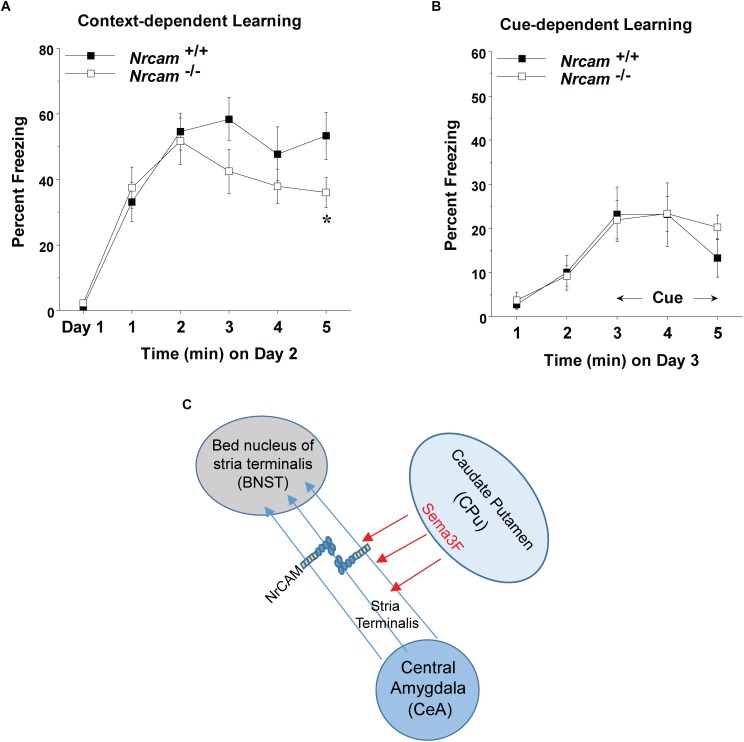
Impaired contextual fear conditioning in NrCAM null mice. **(A)** Baseline levels of freezing behavior before shock exposure were determined on day 1 (the training day). Contextual learning was evaluated on day 2 of testing. **(B)** Cued learning was determined on day 3, using an 80-decibel acoustic stimulus in the last 3 min of the 5-min session. Data shown represent means (+SEM), ^∗^*p* = 0.0349). **(C)** Hypothetical model showing axonal projections of the ST between the CeA and BNST. Fasciculation of axons expressing NrCAM in the ST may be mediated by repulsion from Sema3F secreted by cells in the caudate putamen (CPu), or through homophilic NrCAM-NrCAM adhesion between axons.

Dysconnectivity between the CeA and BNST may disrupt limbic circuits involved in regulation of behavioral responses such as fear and sociability (Lebow and Chen, 2016). male NrCAM null mutant mice exhibit sociability deficits, cognitive inflexibility, and hyper-responsiveness to sensory stimuli but fear conditioning was not tested (Moy et al., 2009b).To determine if loss of NrCAM affected behavior responses involving limbic function, cued (tone-shock) and contextual fear conditioning were analyzed in adult WT and NrCAM null male mice. We analyzed male WT and NrCAM null mutant mice in fear conditioning, because only males showed impairments in sociability, reversal learning, and sensory gating (Moy et al., 2009b). Moreover, ASD is more common in males than females across age groups (Hull et al., 2017). We tested both cued and context-dependent fear conditioning in well-characterized learning paradigms (Huang et al., 2013; Zhu et al., 2014). Cued fear conditioning involves circuits in the CeA, basolateral amydgala, and periaqueductal gray of the brainstem, whereas contextual fear conditioning is mediated through circuitry involving the CeA and BNST, as well as the hippocampus and mPFC (Stamatakis et al., 2014). Deletion of NrCAM in NrCAM mutant mice led to selective deficits in context-dependent learning, measured by percent freezing over a 5 min period on day 2 following the training day 1 [genotype *x* time interaction, *F*(5,105) = 2.50, ^∗^*p* = 0.0349] (Figure 3A). In contrast, both the WT and mutant mice showed similar levels of cue-dependent learning measured on day 3 (Figure 3B). No group differences were observed in baseline levels of freezing (measured as percent time immobile) during the first exposure to the conditioned fear chamber.

## Discussion

In this study we analyzed the expression and function of NrCAM in promoting axon fasciculation in the ST, a key tract connecting the CeA with targets in the BNST. In embryonic mouse brain (E17.5) NrCAM was found to be expressed in developing amygdalar nuclei, including the CeA, during establishment of connectivity with the BNST. Deletion of NrCAM in homozygous null mutant male mice revealed a phenotype of axon defasciculation in the ST, suggesting that NrCAM promotes axon bundling and proper targeting of these projections. Behavioral testing of WT and NrCAM null mice showed that NrCAM loss impaired contextual fear conditioning, a response dependent on CeA-BNST circuitry. These results demonstrate a novel function for NrCAM in development of limbic circuitry.

Neuron-glia-related cell adhesion molecule mediates repellent signaling in axons as an obligate component of the Sema3F holoreceptor complex. As shown in Figure 1A, NrCAM binds the Sema3F co-receptor Npn2 and promotes signaling through the associated PlexA3 subunit. It has been reported that Sema3F transcripts are expressed in the embryonic CPu, which borders the ST, while Npn2 transcripts are present in the CeA and other amygdaloid nuclei (Sahay et al., 2003). Sema3F- and Npn-2-deficient mice display disorganized and defasciculated axons of amygdalar projections to the BNST (Sahay et al., 2003), like the phenotype of NrCAM null mice observed here. Our results showed that NrCAM was localized to fibers in the developing ST (Figure 2C). Accordingly, loss of NrCAM may impair a repellent response of CeA axons to Sema3F, resulting in defasciculation and mistargeting of ST axons, as depicted in Figure 3C. In support of this notion, Sema3F deletion in mice has been associated with abnormalities in fear-related responses in mice (Matsuda et al., 2016). Homophilic adhesion between axons through NrCAM-NrCAM binding could also contribute to ST axon fasciculation. Both mechanisms likely depend on cytoskeletal interactions of the NrCAM cytoplasmic domain with Ankyrin, ERM, and/or PDZ-domain scaffold proteins.

The CeA to BNST projection is topographically specified, such that CeA axons project to anterolateral nuclei in the BNST, whereas medial amygdalar axons project to posterior and anteromedial BNST nuclei (Dong et al., 2001). A limitation of the present work is that axon tracing has not been performed to determine if topographic targeting relies on selective fasciculation of fibers in the ST through NrCAM interactions. Analogously, Sema3A, a closely related secreted Semaphorin, promotes selective fasciculation of olfactory sensory fibers in a pre-target sorting mechanism for olfactory topographic map formation in mice (Imai et al., 2009). Also, axons from the amygdala reach the BNST via two distinct pathways: the ST (dorsal) and ansa peduncularis (ventral) (Dong et al., 2001). The integrity of the ansa peduncularis has not been investigated for an effect of NrCAM deletion.

Lesion and pharmacological studies have indicated that the BNST is involved in the modulation of innate fear responses (Davis et al., 1997; Xu et al., 2012). The present finding that NrCAM null mice exhibit decreased contextual fear conditioning is in accord with altered CeA-BNST connectivity in modulating fear (Amano et al., 2010). In contrast, NrCAM deletion did not affect cued fear conditioning, which relies on connections between the CeA and basolateral amygdala (Stamatakis et al., 2014). Our studies differ in part from Matzel et al. (2008), who reported no effect of NrCAM deletion on cued or contextual fear conditioning using a water licking paradigm. In that study, NrCAM mutant mice were on a hybrid background of Sv129 and outbred Swiss Webster, whereas our mice were predominantly on C57/BL6. Finally, it should be noted that NrCAM loss may disrupt connectivity within other brain regions that contribute to contextual fear conditioning, such as the hippocampus and mPFC. In this regard, the increased dendritic spine density of mPFC pyramidal neurons in NrCAM mutant mice (Mohan et al., 2018) might impact “top-down” regulation of fear conditioning responses.

In summary, the present study demonstrates the novel finding that NrCAM is expressed in the embryonic amygdala-BNST pathway and is required for proper axon fasciculation of the ST. Mistargeting of axons in the CeA-BNST projection may contribute to impaired contextual fear conditioning in mice with loss-of-function mutations in NrCAM.

## Author Contributions

VM and JG performed NrCAM expression experiments and analysis of axon projections. Mouse behavioral testing was performed in the UNC Mouse Behavioral Phenotyping Core. PM designed and analyzed the experiments, and wrote the manuscript with VM.

## Conflict of Interest Statement

The authors declare that the research was conducted in the absence of any commercial or financial relationships that could be construed as a potential conflict of interest.
